# Relationship between family functioning and affiliate stigma in parents of children with autism spectrum disorder in China: the mediating role of positive aspects of caregiving

**DOI:** 10.3389/fpsyt.2025.1613340

**Published:** 2025-07-21

**Authors:** Xiaoyan Du, Xiangdan Su, Dandan Ding, Yiru Zhu, Yongrong Sun, Miaomiao Wang, Yang Xiao, Haiping Xu

**Affiliations:** Department of Child Development and Behavior, The Third Affiliated Hospital of Zhengzhou University, Zhengzhou, China

**Keywords:** autism spectrum disorder, parents of children with ASD, caregiver, family functioning, affiliate stigma, positive aspects of caregiving, mediating effect

## Abstract

**Objective:**

The present research was conducted to examine whether family functioning is related to affiliate stigma in Chinese caregivers of children with autism spectrum disorder (ASD) and how positive aspects of caregiving affect this relationship.

**Methods:**

Two hundred and six caregivers of children with ASD were investigated using the Family APGAR scale, the Affiliate Stigma scale, and the Chinese version of the Positive Aspects of Caregiving scale. The statistical methods of Pearson correlation analysis and mediation effect analysis were used to statistically analyze the relationship between family functioning, affiliate stigma and positive aspects of caregiving.

**Results:**

The results indicated that family functioning and positive aspects of caregiving were negatively associated with affiliate stigma and that family functioning was positively correlated with positive aspects of caregiving. Furthermore, positive aspects of caregiving partially mediated family functioning and affiliate stigma.

**Conclusion:**

These findings suggest that family functioning can directly influence the affiliate stigma of caregivers of children with ASD and indirectly influence affiliate stigma through positive aspects of caregiving.

## Introduction

Autism spectrum disorders (ASDs), commonly referred to as autism, are neurodevelopmental conditions present at birth that are characterized by significant social deficits, delayed language acquisition, restricted interests, and repetitive behaviors (1). The global prevalence of ASD is steadily increasing and is currently estimated to be 1%–2% (2). In China, the prevalence of ASD is 1%, including over 13 million individuals (3, 4). This makes ASD one of the fastest growing neurodevelopment disorders in terms of incidence rate (2, 5). Currently, there is no targeted treatment for ASD, with long-term interventional therapy serving as the primary approach (6, 7). Family caregivers assume a central role in the care and support of individuals with ASD (8, 9). Unfortunately, the focus on ASD often leads to the mental well-being of the caregivers being overlooked. Research indicates that parents of children with autism commonly experience prolonged periods of suboptimal mental health throughout their child’s diagnosis and treatment (10, 11).

### Affiliate stigma

Affiliate stigma (AS) linked to ASD significantly affects the physical and mental well-being of primary caregivers (12, 13). This AS, also known as stigmatization, manifests as self-isolation and group avoidance due to the discrimination, slander, and exclusion that result from close associations with stigmatized individuals or communities (14). Public stereotypes (15), a lack of social support (16), financial stress (17), and uncertainty about the effectiveness of treatment (7) make it more difficult for children with ASD to improve over time. All of these factors reinforce stigmas and lead to negative feelings among caregivers. Research indicates that caregivers of children with ASD who experience high levels of stigma tend to exhibit social avoidance and withdrawal behaviors (12, 13, 18), including concealing their child’s condition. Such behavior negatively impacts their attitude toward their child and caregiving practices (19, 20), consequently affecting intervention management (12). Therefore, scholars emphasize the importance of implementing targeted interventions for caregivers of children with ASD to mitigate stigma and improve care quality (21, 22).

### Family functioning

Symptom severity in children with ASD is correlated with parents’ mental health, quality of life, and caregiving burden (16, 23–25). Consequently, caregivers of children with ASD commonly seek support, either internally or externally (16, 26). Family support is a readily accessible resource for both patients and caregivers. Previous studies have shown that certain characteristics of the family system are essential for a child’s healthy development (27). Hence, family units are of paramount importance in the context of patients and their caregivers (28, 29). Empirical evidence has demonstrated that a robust family support system can enhance psychological resilience. Family functioning (FF) can enhance caregivers’ self-efficacy and empower them to effectively take on life challenges effectively (30, 31). FF encompasses various forms of support, including physical, financial, emotional, and psychological assistance from family members, which manifests as satisfaction with family dynamics (32). Quality family care not only contributes to patients’ recovery, mental well-being, and healthy habits but also benefits caregivers’ physical, psychological, and overall quality of life (33, 34). Lovell and Wetherell (35) reported that parents of children with ASD who perceived greater familial support tended to report reduced levels of shared stigma. Similarly, as Meulien and Baghdadli (36) noted, caregivers’ perceptions of shared stigma are influenced by the presence of a robust support system for children with ASD. Among them, family, friends, and professional support are important protective factors to prevent stigmatization.

### Positive aspects of caregiving

Family caregivers play crucial roles in the treatment and rehabilitation of ASD children. The well-being and health of caregivers, including their quality of life and physical and mental health, are increasingly under scrutiny because of the significant responsibilities and high costs associated with long-term care for children with ASD (25, 37). A substantial care burden often leads to negative emotions and feelings among caregivers (10, 12). Nevertheless, the emergence of positive psychology has shed light on the fact that caregiving is not linked solely to adverse outcomes; caregivers can also derive positive emotions, subjective benefits, and satisfaction from the caregiving process (38). The positive aspects of caregiving (PAC) include the beneficial impact of caregiving on caregivers, including a sense of personal achievement and enhanced family unity (38). Research indicates that the positive aspects experienced by caregivers can enhance their well-being and satisfaction (39), decrease their stress level and depressive symptoms (40), bolster their commitment to caregiving, and enhance the quality of the care provided (41). The literature suggests a link between FF and PAC (42, 43). Effective FF can strengthen family bonds, facilitate caregivers’ receipt of increased care and affection from family members, and provide caregivers with greater support and encouragement. Increased encouragement and support can effectively mitigate internal conflicts resulting from traumatic events, facilitate positive changes, reduce negative emotions, and foster the development of positive aspects (44).

### Kramer’s caregiver adaptation model

Kramer’s caregiver adaptation model (45) offers a theoretical framework for examining the interplay between FF, PAC experiences, and AS. This model underscores the significance of caregivers’ external (e.g., FF) and internal resources (e.g., coping strategies) in shaping their caregiving perceptions (e.g., positive aspects and burden). Positive aspects of FF are connected to healthy ways of coping (such as feeling more confident in one’s ability and adjusting to new roles), which affect health and behavior (such as noticing discrimination less frequently and growing as a person). With the help of the caregiver adaptation model, PAC may play a role in the link between FF and AS that caregivers of people with ASD experience. However, to our knowledge, few studies have focused on the relationships among FF, PAC, and AS in patients with ASD.

In summary, this study aimed to examine (a) the relationship between FF and AS and (b) the mediating role of PAC in Chinese caregivers of children with ASD. We used measures to assess the FF, PAC, and AS levels among caregivers of children with ASD and conducted a mediation analysis to examine how PAC mediates the relationship between FF and AS.

## Materials and methods

### Participants

Two hundred and six caregivers of children with ASD participated in this study. The caregivers were recruited from a hospital in the city of Zhengzhou, China. All of the children were diagnosed with ASD and received rehabilitation at this hospital. Inclusion criteria for participants: 1) the main caregiver of the child, the time of care≥6 months; 2) informed consent, voluntary participation; 3) the child under the care of the U.S. Diagnostic and Statistical Manual of Mental Disorders, fifth edition of the diagnosis of ASD; 4) literacy, understanding the purpose of the questionnaire survey and the significance. Exclusion criteria: 1) the caregiver has a major physical illness or a history of mental illness; 2) there are other serious negative events in the caregiver’s family; 3) the child suffers from other serious organic diseases. This study was approved by the Ethics Committee of The Third Affiliated Hospital of Zhengzhou University (2022-391-01).

The ages of the caregivers ranged from 20 to 67 years (M = 34.13, SD = 8.15). Most of the caregivers were female (68.0%). Half of the researchers do not have a university degree. The children were between 2 and 9 years of age (Mage = 4.72, SD = 1.54). The patients’ demographic characteristics are shown in **Table 1**.

**Table 1:** Demographic characteristics of the participants (*n* = 206).

Variable		Frequency (f)	Percentage (%)
Parent’s gender	Male	66	32.0
	Female	140	68.0
Residence	Rural	90	43.7
	City	116	56.3
Parent’s education level	Middle school and below	56	27.2
	High school degree	47	22.8
	University degree	91	44.2
	Master’s degree and above	12	5.8
Marital status	Married	194	94.2
	Divorced	7	3.4
	Remarried	5	2.4
Religious beliefs	Yes	12	5.8
	No	194	94.2
Autism severity	Mild	42	20.4
	Moderate	102	49.5
	Severe	62	30.1
Intervention time for pediatric patients (months)	<12	116	56.3
	12-24	44	21.4
	>24	46	22.3

### Measures

#### Family APGAR scale

Chinese caregivers for children with ASD use the APGAR scale to measure their FF levels (46). The method includes five items and uses a 3-point scale from 0 (“rarely”) to 2 (“often like this”). Sample items include “When I encounter problems, I can obtain satisfactory help from my family” and “I am happy with the way my family discusses things with me and shares problems.” The total score ranges from 0–10 points, with 0–3 points indicating that family function is severely impaired, (i.e., low family care), 4–6 points indicating that family function is moderately impaired, (i.e., medium family care), and 7–10 points indicating that family function is good, (i.e., high FF). In this study, the Cronbach’s coefficient for this scale was 0.925.

#### Affiliate stigma scale

Chinese caregivers of children with ASD measure AS using the ASS (47). The ASS includes 18 total items derived from four dimensions: negative emotional cognition, social anxiety avoidance, alienation from the stigma, and discrimination experience. Sample items are as follows: “I was emotionally troubled because I had an autistic child at home” and “Having a child with autism has a negative impact on me.” Items are scored on a 4-point scale from 1 (“strongly disagree”) to 4 (“strongly agree”). The average score of all of the items is determined, and higher average scores indicate higher stigma levels. In this study, the Cronbach’s coefficient of the scale was 0.959.

#### The Chinese version of the positive aspects of caregiving scale

The Chinese version of the Positive Aspects of Caregiving scale is used to measure PAC in Chinese caregivers of children with ASD (48, 49). Sample items are as follows: “Makes me feel useful” and “Makes me feel needed.” The scale includes two dimensions and nine items. The items are rated on a 5-point scale from 1 (“strongly disagree”) to 5 (“strongly agree”), and higher scores indicate that the caregiver has more positive aspects. The Cronbach’s coefficient of the scale was 0.921.

### Procedures

With assistance from the department and the hospital, the research team contacted caregivers of children with ASD who were interested in participating in this study. Before the survey began, members of the research team introduced the study objectives and the participants’ rights to each parent who completed the questionnaire. All of the participants provided informed consent to participate in this study. This study was approved by the hospital ethics committee.

### Data analysis

Statistical analyses of the data were conducted using SPSS software (version 23.0). At this stage, first, whether the data complied with normality assumptions was examined. The descriptive statistics and normality values of the data are presented in **Table 2**. According to the results in **Table 2**, the skewness and kurtosis values of the data were within acceptable ranges (50). Additionally, the data were examined for multicollinearity. Multicollinearity was assessed using variance inflation factors (VIFs), with all values less than 5, suggesting no significant multicollinearity concerns (51). Next, we conducted Pearson’s correlation analyses to examine the relationships among PAC, FF, and AS in caregivers of children with ASD. The highest value obtained from the correlation analysis between the variables was 0.443. Therefore, it was possible to say that there was no multicollinearity problem between the variables. We then used the PROCESS macro in SPSS to examine the mediating role of PAC in the relationship between FF and AS. The PROCESS macro uses a path analysis modeling tool based on regression to assess the direct and indirect effects of variables (52). In accordance with the relevant literature, we performed mediation analyses using Model 4 with 5000 bias-corrected bootstrap samples.

**Table 2:** Descriptive statistics and coefficients of skewness-kurtosis (*n* = 206).

Variables	N	Minimum	Maximum	Skewness	Kurtosis
PAC	206	9	45	-0.303	-0.120
FF	206	1	10	-0.265	-0.802
AS	206	18	72	-0.243	0.090

## Results

### Descriptive and correlational analyses

The mean total scores and correlations among the three scales are presented in **Table 3**. The results indicated that PAC was negatively correlated with AS (*r* = -0.44, *P* < 0.001) and positively correlated with FF (*r* = 0.25, *P* < 0.001), indicating that caregivers of children with ASD reported high levels of PAC if they had high FF levels and low AS levels. In addition, FF was negatively correlated with AS (*r* = -0.39, *P* < 0.001). This result indicates that high FF levels in parents are associated with lower AS levels.

**Table 3:** Descriptive and correlational analyses (*n* = 206).

Variable	Mean	SD	PAC	FF	AS
PAC	30.77	8.51	–		
FF	5.83	3.11	0.245***	–	
AS	46.77	12.48	-0.443***	-0.390***	–

***P<0.001.

### The mediating role of PAC in the relationship between FF and AS

**Figure 1:**
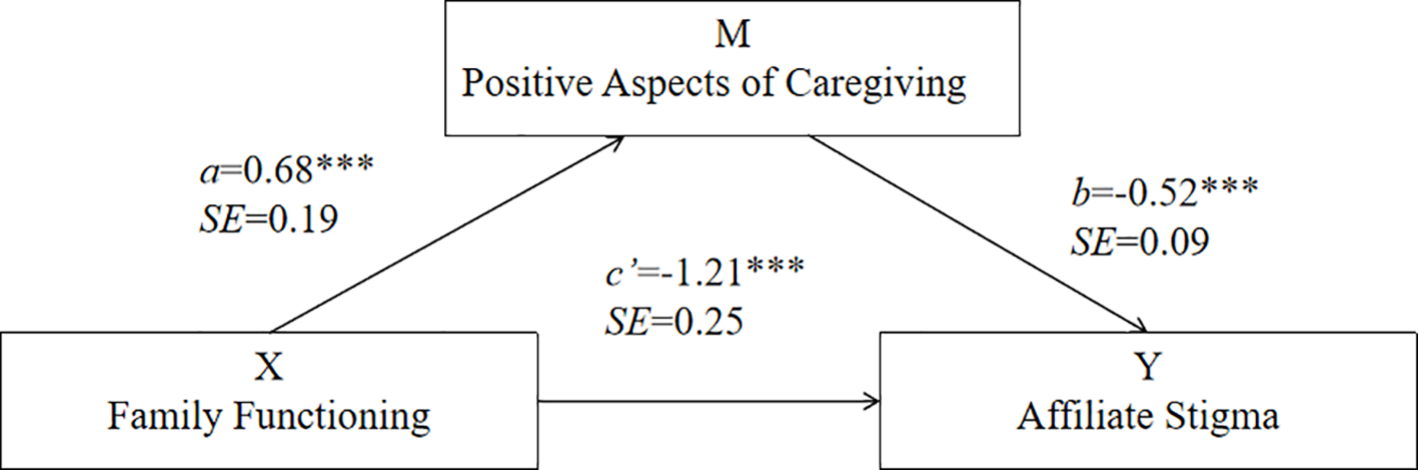
The mediation model for the effect of PAC in the relationship between FF and AS. ***P<0.001.

To test whether PAC mediates the relationship between FF and AS, we performed a mediation analysis with FF as a predictor, PAC as a mediator, and AS as the dependent variable. The mediation model is illustrated in **Figure 1**. The results revealed that the effect of FF on the PAC was -0.68 (SE = 0.18, *t* = 3.67, *P* < 0.001, 95% CI: 0.32 to 1.05). Furthermore, the effect of PAC on AS was -0.52 (SE = 0.09, *t* = -5.68, *P* < 0.001, 95% CI: -0.69 to -0.34). Importantly, FF had a total effect on AS of -1.56 (SE = 0.26, *t* = -6.04, *P* < 0.001, 95% CI: -2.07 to -1.05). Specifically, the indirect effect of FF on AS was -0.35 and significantly different from zero (95% CI: -0.62 to -0.13), whereas the direct effect was -1.21 (SE = 0.24, *t* = -4.87, *P* < 0.001, 95% CI: -1.70 to -0.72). This model explained 13.68% of the variance in AS (F = 37.22, *P* < 0.001). Overall, these results suggest that PAC partially mediates the relationship between FF and AS.

## Discussion

### Clinical and intervention implications

This study aimed to investigate the relationship between FF and AS among caregivers of Chinese children with ASD and the mediating role of caregivers’ PAC. First, the study’s results demonstrated a negative relationship between FF and AS. Specifically, caregivers of children with ASD who perceived stronger FF had lower AS, which is consistent with previous research (13, 31, 53). From a theoretical perspective, FF, as an external resource in the caregiver adaptation model, can make important contributions to positive psychological adaptation (42), allowing caregivers to maintain an optimistic psychological state, which reduces their AS (36). FF reflects a caregiver’s access to family support. For caregivers of children with ASD, FF is an important factor in helping them cope with the stress of caring for their children and promoting their children’s recovery, which helps reduce their feelings of AS (28). Clinical staff should monitor family caregivers of children with ASD; encourage family members to maximize the supportive function of the family system; and ensure that caregivers have more emotional, value affirmation, and financial support from the family. Factor et al. (54) reported that research has broadly shifted to focusing on families, highlighting the influence of family support and functioning for families of children with ASD in everyday life and in intervention procedures. There have been intervention studies on the family functioning of caregivers for children with ASD, and the relevant research results can provide a reference for us to conduct similar studies in the future. Peng et al. (55) implemented a family task intervention program to enhance the family functioning of caregivers for children with autism, including (1) medical staff helping caregivers gain relevant health experience, skills, material resources, and services for managing children with autism and (2) by understanding the family structure and general condition of the sick child, medical staff can help caregivers recognize the importance of internal and external support within the family and learn how to cope with illness and emotional problems in daily life. They can provide psychological support and assist in obtaining social assistance. Sabanciogullari and Yildırım (56) conducted a 10-week group counseling and education program (GCEP) for parents with ASD. GCEP can help parents with ASD raise awareness and provide them with social and psychological support, which can help them clarify family relationships, care, and responsibilities for children with ASD. The results showed that GCEP can effectively improve psychological resilience and family functioning.

### The need for PAC support

Second, the results indicate a significant negative correlation between caregivers’ PAC and AS. Specifically, high PAC levels can lessen the negative feelings associated with AS. This finding is consistent with those from previous studies (57, 58). PAC is the positive experience that caregiving brings to the caregiver’s life, which is reflected mainly in having a strong ability to cope with stress and an improved subjective health status (38, 59). For Chinese families in which a member has ASD, the prejudice and stereotyping of autism by social groups, along with the discrimination and rejection of children with autism and their caregivers, leads to a strong sense of AS among caregivers (12, 13, 21). Most caregivers adopt ineffective coping strategies (e.g., reducing socialization and avoiding interpersonal interactions) to cope with AS (35, 36). However, some studies have shown that caregivers of children with ASD seek supportive resources in the process of caring for their children and derive benefits from their children’s recovery, such as knowledge about autism recovery; improved caregiving skills; and positive feelings, such as perceived value, meaning of life, and self-growth (60, 61). Therefore, providing a multitude of support resources to caregivers of children with ASD can increase PAC and reduce AS. A literature review found that, at present, there are few intervention studies on the positive feelings of caregivers for children with ASD, mainly through qualitative research to understand the positive aspects of caregivers raising ASD children (62). With respect to relevant research results, we can gradually explore the role of positive psychological intervention, focused solution model intervention and caregiver network intervention models based on PAC models in improving the positive feelings of caregivers for children with ASD (63–66).

### Theoretical implications

Third, and most importantly, the results suggest that PAC mediates the relationship between FF and AS. These findings support the caregiving adaptation model. FF is an important external resource for caregivers in caring for children with ASD, enabling them to receive more support and encouragement, which alleviates negative emotions, enhances PAC formation, and buffers caregiver stress (28). In addition, the caregivers’ PAC indicates an individual’s optimistic view of life events, and PAC in the caregiving process can reduce caregiver stress, making caregivers more inclined to adopt a positive and optimistic attitude toward parenting children with autism (37). A previous study indicated that the enhancement of positive feelings in caregivers prevents the accumulation of internalized negative experiences and emotions and reduces AS (57). Therefore, in order to better alleviate the AS associated with caring for children with ASD, it is necessary to give full play to the role of FF, help caregivers discover PAC in the caregiving process, and thereby weaken negative emotions.

## Limitations

In conclusion, the present study validated the relationships among FF, PAC, and AS among caregivers of children with ASD, but these results should be interpreted with caution. This study has several limitations. First, the participants in this study were recruited from only one hospital, and the sample size was relatively small; therefore, the generalizability of the findings must be validated by increasing the sample size. Second, this was a cross-sectional survey study and all of the data were based on caregivers’ self-reports, which precluded us from interpreting the causal relationships among FF, PAC, and AS among caregivers of children with ASD. Third, the ages of the children with ASD included in this study ranged from 2 to 9 years old. Given the changes in social demands and needs as children enter adolescence and adulthood, future studies should examine whether the same relationships are observed in older age groups.

Therefore, in the future, we can perform more qualitative and longitudinal studies to better confirm the links among FF, PAC, and AS. We can also identify scientifically sound ways to help caregivers of children with ASD improve their FF and PAC, which will lower their AS. Furthermore, children with ASD of different age groups should be selected and included in the sample, and the current status of their caregivers’ FF, PAC and AS, influencing factors and the relationships among the three, should be investigated to formulate targeted intervention plans. Future research should address these issues.

## Conclusion

This study explored the relationships among FF, PAC, and AS among caregivers of children with ASD in China. These findings support a relationship between FF and AS. In addition, caregivers’ PAC mediated this relationship. The study results emphasize the critical role of FF and PAC in enhancing psychological resources and alleviating AS associated with caregivers of children with ASD. These findings suggest that interventions aimed at strengthening FF and enhancing PAC may be effective strategies for alleviating AS associated with caregivers of children with ASD. Healthcare professionals should consider incorporating FF and PAC into support programs for these caregivers. These findings may provide valuable information on how to reduce AS among caregivers of children with autism in China. Healthcare professionals should adopt family-centered interventions with the participation of family members to help caregivers of children with ASD establish a good family support system, promote PAC enhancement, and reduce AS so parents can adjust their mindsets to actively participate in the rehabilitation process of their children and better integrate into society.

## Data Availability

The raw data supporting the conclusions of this article will be made available by the authors, without undue reservation.
